# Percutaneous left atrial appendage occlusion in a frail, high-risk, octogenarian patient population, after having undergone transcatheter aortic valve implantation

**DOI:** 10.1186/s12872-022-02786-4

**Published:** 2022-08-02

**Authors:** Ioannis Drosos, Roberta De Rosa, Sebastian Cremer, Philipp C. Seppelt, Katrin Hemmann, Jana Oppermann, Recha Blessing, Silvia Mas-Peiro, Mariuca Vasa-Nicotera, Andreas M. Zeiher, Zisis Dimitriadis

**Affiliations:** 1 Division of Cardiology, Department of Medicine III, University Hospital Frankfurt, Goethe University Frankfurt Am Main, Theodor-Stern-Kai 7, 60590 Frankfurt am Main, Germany; 2 Center of Cardiology, Cardiology 1, University Medical Center Mainz, Mainz, Germany; 3 German Center for Cardiovascular Research (DZHK), Berlin, Germany; 4 Cardiopulmonary Institute (CPI), Frankfurt, Germany

**Keywords:** Left atrial appendage occlusion, Atrial fibrillation, Bleeding risk, Frail, Elderly

## Abstract

**Background:**

Percutaneous left atrial appendage occlusion (LAAO) represents an alternative stroke prevention method in patients with atrial fibrillation and an increased bleeding risk, chronic kidney disease or contraindications to oral anticoagulants. Aim of our study was to evaluate the feasibility and safety of percutaneous LAAO in high-risk, frail patients having undergone transcatheter aortic valve implantation (TAVI).

**Methods:**

Thirty-one patients having undergone TAVI and scheduled for LAAO were prospectively included in our study.

**Results:**

Implantation was successful in 29 of 31 cases (93.5%).There were no patients that developed a major acute cardiovascular event, stroke, or device dislocation/embolization. There was a single case of major bleeding (3.2%) and 3 cases of acute kidney injury (9.7%). At 3 months, no patients experienced a stroke, one patient had a device-related thrombus (3.4%), one patient showed a significant peri-device leak, and one patient had a persistent iatrogenic atrial septal defect.

**Conclusions:**

Our study shows that percutaneous LAAO may represent a feasible alternative strategy for stroke prevention, that can be safely performed in high-risk, multimorbid patients with high bleeding risk or contraindications to oral anticoagulation.

## Introduction

Atrial fibrillation (AF) represents the most common persistent arrythmia worldwide, posing a significant burden for health systems around the world [1]. The prevalence of AF, at present ranging between 2 and 4%, is expected to rise, mainly because of increasing age and risk factors associated with AF, such as heart failure, coronary artery disease, hypertension, and diabetes mellitus [1]. Stroke prevention has become a major issue of patient management, as patients with AF have a five-fold increased risk of stroke compared with patients without AF [2]. Newer, non-vitamin K antagonist oral anticoagulants (NOACs) represent a milestone in stroke prevention for patients with AF. The importance of bleeding risk assessment when initiating antithrombotic therapy cannot be overemphasized. According to current guidelines and consensus statements, bleeding risk assessment using the HAS-BLED score is important for all patients with AF and non-drug stroke prevention options should be considered in cases of patients with an increased bleeding risk or absolute contraindications to oral anticoagulation (OAC) [2, 3]. Percutaneous left atrial appendage occlusion (LAAO) represents an alternative strategy for stroke prevention. Safety, efficacy, and non-inferiority of LAAO compared to vitamin K antagonists and NOACs in patients with high bleeding risk have been shown in randomized clinical trials and meta-analyses [4–7]. Nowadays, LAAO represents a promising alternative for elderly patients with increased bleeding risk and various comorbidities.

Such a patient group is well represented by patients undergoing transcatheter aortic valve implantation (TAVI). Until recently, TAVI was recommended for older patients and patients who are high-risk or not suitable for surgical aortic valve replacement. This has been changed in current guidelines, as TAVI can now be considered also in patients with moderate or low risk after careful assessment of individual clinical, anatomical and procedural characteristics [8]. However, TAVI still represents in clinical practice the preferred method in patients in whom prosthetic valve durability is of low priority (older patients ≥ 75 years), or patients who are inoperable or high-risk for surgery. These patients are, mostly, elderly with multiple comorbidities, such as heart failure, diabetes mellitus, chronic lung disease and chronic kidney disease. Scores used to assess operative mortality, such as the EuroSCORE II, have some parameters in common with the HAS-BLED score, such as age and renal impairment. Several patients with AF undergoing TAVI have an increased HAS-BLED score, former bleeding events, chronic kidney disease or other contraindications to OAC. This patient collective represents a high-risk patient group not only for surgical, but also for interventional procedures, percutaneous LAAO itself being one of them.

As literature concerning percutaneous LAAO in frail, high-risk patients with high bleeding risk is sparse, we aimed at investigating feasibility and safety of percutaneous LAAO in such a patient collective having undergone TAVI.

## Materials and methods

### Study patients

We prospectively assessed all patients with atrial fibrillation having undergone TAVI that were planned to undergo a percutaneous LAAO in our department, between January 2020 and May 2021. Decision to perform LAAO was taken during the hospital stay after the TAVI procedure and percutaneous LAAO was scheduled 2–3 months after discharge from the hospital. All patients undergoing TAVI had severe aortic stenosis or regurgitation. Our center performs approximately 300–350 TAVI procedures per year. Decision to perform TAVI was met in an interdisciplinary ‘Heart Team’, as recommended by guidelines. LAAO was performed in patients with a history of bleeding, high bleeding risk as assessed by HAS-BLED score, and chronic kidney disease with a relative or absolute contraindication for NOACs (glomerular filtration rate—GFR < 15 ml/min/1,73 m^2^, or “borderline” GFR, or need for dialysis).

Our study complied with the declaration of Helsinki and its later amendments and was approved by the Ethics Committee of the University Hospital of Frankfurt (approval number #20-993). All patients gave their written informed consent prior to their inclusion in the study. We collected data regarding medical history, medication, risk scores, device used, procedure details, peri-interventional results, and complications, as well as follow-up data at 3 months. Major adverse cardiac events (MACE) were defined as myocardial infarction, non-fatal stroke, or cardiovascular death. Major bleeding was defined as a bleeding type 2–4 according to the VARC-3 consensus criteria [9]. Acute kidney injury was defined according to KDIGO Clinical Practice Guideline for Acute Kidney Injury [10].

### Percutaneous LAAO procedure

Preparation for percutaneous LAAO was performed according to standard operating procedures of our department. All patients underwent transesophageal echocardiography (TEE) before the procedure, in order to detect possible thrombi in the left atrial appendage (LAA) and perform measurements of the LAA. Measurements were performed according to the instructions of the manufacturer of the occlusion device used in each case. Either local pharyngeal anesthesia alone or a combination of the latter with conscious sedation using propofol or midazolam were used for the TEE.

LAAO was performed in the catheterization laboratory by two interventional cardiologists. The cardiologist leading the implantation had an experience of at least 50 procedures per year. The decision about the device type was made by the interventional cardiologist leading the procedure, after reviewing the transesophageal echocardiogram from the day before the procedure. Two different LAA occlusion devices were used, namely WATCHMAN FLX™ (Boston Scientific) and Amplatzer™ Amulet™ (Abbott). Conscious sedation with propofol infusion was favored in all cases. Femoral venous access after local anesthesia was obtained and 5000 international units of unfractionated heparin were administered. The whole procedure was performed under TEE guidance. An additional dose of 5000 international units of heparin was administered after transseptal puncture. This second dose was reduced in patients with a body weight < 70 kg and increased in patients with a body weight > 100 kg. Intravenous heparin was administered again, guided by activated clotting time, only when the interventional procedure lasted more than 30 min, aiming at an activated clotting time of > 250 s. LAA dimensions were once more measured using TEE, before choosing device size. After implantation, stable device position was confirmed using the so-called “tug test” (pulling of the occluding device) twice. A transthoracic echocardiography exam was performed 2 h after the intervention to detect possible device dislocation or pericardial effusion. All patients remained on the regular ward for at least two nights after the procedure, according to the standard operating procedures of our department. Clinical inspection of the puncture site (femoral vein) and a transthoracic echocardiography examination were performed on the first postinterventional day. If no complications were detected, patients were discharged on day 2 after the procedure. After LAAO, patients received a dual antiplatelet therapy with aspirin and clopidogrel for 3 months, followed by lifelong aspirin monotherapy. A follow-up examination including transthoracic and transesophageal echocardiography was performed at 3 months as part of the standard follow-up of patients undergoing LAAO in our department.

### Data presentation—statistical analysis

Quantitative data are presented as mean ± standard deviation and categorical data as frequency and percentage. Frequencies were calculated using Microsoft Excel and descriptive statistics using GraphPad Prism version 8.0.

## Results

### Study population

**Fig. 1:**
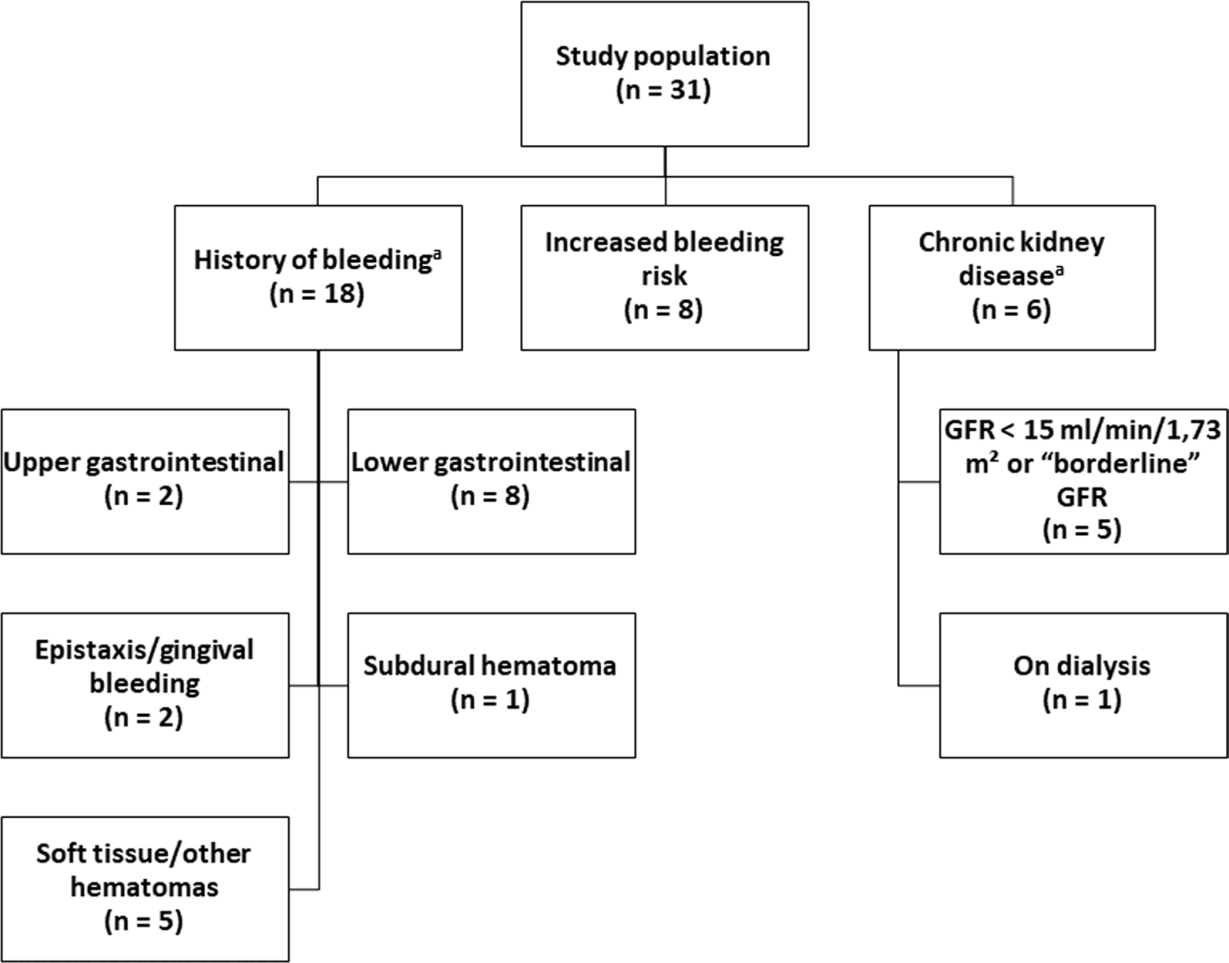
Indications for LAAO in the study population. ^a^ One patient falls under both groups, having a history of bleeding and chronic kidney disease

Thirty-one consecutive patients (20 male, 11 female; mean age, 82.1 ± 4.4 years) having undergone TAVI and about to undergo percutaneous LAAO were included in our study. Demographics and clinical characteristics of the study population are summarized in Table 1 and Table 2. All patients had been diagnosed with atrial fibrillation and were under OAC before undergoing TAVI. A vast majority of included patients had at least one cardiovascular risk factor or manifestation of cardiovascular disease. Included patients had an average operative mortality of 6.8 ± 4.7% as assessed by EuroSCORE II, which confirms that patients had an increased comorbidity burden. Indications for LAAO are summarized in Fig. 1.

**Table 1:** Basic characteristics of the study population

Parameter	Study population (n = 31)
*Demographics*	
Age, (years)	82.1 ± 4.4
Gender, (male/female)	20 / 11
*Anthropometrics*	
Body weight, (kg)	81.5 ± 20.1
Body height, (cm)	163.0 ± 21.4
Body-mass-index, (kg/m^2^)	27.8 ± 3.7

Values are expressed as mean ± standard deviation

**Table 2:** Clinical characteristics and comorbidities of the study population

Parameter	Study population (n = 31)
*Risk scores*	
CHA_2_DS_2_-VASc	5.0 ± 1.2
HAS-BLED	4.0 ± 0.7
EuroSCORE II	6.8 ± 4.7
*Cardiovascular risk factors*	
Diabetes mellitus, [n (%)]	11 (35.5%)
Hypertension, [n (%)]	30 (96.8%)
Hyperlipidemia, [n (%)]	24 (77.4%)
Smoking, [n (%)]	3 (9.7%)
Family history, [n (%)]	2 (6.5%)
Obesity, [n (%)]	10 (32.3%)
*Cardiovascular disease*	
Atrial fibrillation, [n (%)]	31 (100.0%)
Paroxysmal, [n (%)]	17 (54.8%)
Persistent, [n (%)]	9 (29.0%)
Permanent, [n (%)]	5 (16.1%)
Coronary artery disease, [n (%)]	26 (83.9%)
Myocardial infarction, [n (%)]	8 (25.8%)
PCI, [n (%)]	11 (35.5%)
CABG, [n (%)]	4 (12.9%)
PAD or carotid artery disease, [n (%)]	8 (25.8%)
Heart valve disease (other than aortic), [n (%)]	24 (77.4%)
Stroke, [n (%)]	5 (16.1%)
Aortic aneurysm, [n (%)]	2 (6.5%)
PE or DVT, [n (%)]	2 (6.5%)
Heart failure, [n (%)]	12 (38.7%)
*Comorbidities*	
Autoimmune diseases, [n (%)]	1 (3.2%)
COPD, [n (%)]	4 (12.9%)
Chronic kidney disease, [n (%)]	21 (67.7%)
Liver disease, [n (%)]	1 (3.2%)
*Devices*	
Pacemaker, [n (%)]	6 (19.4%)
ICD, [n (%)]	2 (6.5%)
CRT, [n (%)]	0 (0.0%)
*Biochemical parameters*	
Hemoglobin, (g/dL)	11.4 ± 2.5
Creatinine, (mg/dL)	1.7 ± 1.3
GFR, (ml/min/1,73 m^2^)	43.6 ± 16.9
NT-proBNP, (pg/mL)	4,900 ± 7,272

Values are expressed as mean ± standard deviation for continuous variables and as number of patients and % for categorical variables

*PCI* percutaneous coronary intervention; *CABG* coronary artery bypass graft surgery; *PAD* peripheral arterial disease; *PE* pulmonary embolism; *DVT* deep vein thrombosis; *COPD* chronic obstructive pulmonary disease; *ICD* implantable cardioverter-defibrillator; *CRT* cardiac resynchronization therapy; *GFR* glomerular filtration rate

### LAAO procedure

As already mentioned, two different LAAO devices were used in all patients. WATCHMAN FLX™ (Boston Scientific) and Amplatzer™ Amulet™ (Abbott). An Amplatzer™ Amulet™ occlusion system was used in most cases (58.1% Amplatzer™ Amulet™ vs. 35.5% WATCHMAN FLX™). Procedure duration ranged between 22 and 100 min, with a mean duration of 55.7 ± 20.0 min. Mean fluoroscopy time was 12.4 ± 4.7 min and mean radiation dose 30.0 ± 85.2 Gy x cm^2^. Main peri-interventional guiding tool was TEE with no contrast medium at all being used in 21 of 31 cases (67.7%). TEE was not directly possible in one case because of a hypopharyngeal diverticulum, so that endoscopic guidance was needed to achieve transesophageal ultrasound probe insertion. In one case (3.2%) the occlusion device size had to be changed to a smaller size during the intervention. The reasons to decrease size was inadequate device stabilization and protrusion in the left atrium. Peri-procedural details are summarized in Table 3.

**Table 3:** Peri-procedural details

Parameter	Study population (n = 31)
Procedure duration, (minutes)	55.7 ± 20.0
Fluoroscopy time, (minutes)	12.4 ± 4.7
Radiation dose, (Gy x cm^2^)	30.0 ± 85.2
Contrast medium volume, (ml)	12.3 ± 20.8
*Occlusion device and size*	
WATCHMAN FLX™, [n (%)]	11 (35.5%)
20 mm, [n (%)]	1 (3.2%)
24 mm, [n (%)]	4 (12.9%)
27 mm, [n (%)]	5 (16.1%)
35 mm, [n (%)]	1 (3.2%)
Amplatzer™ Amulet™, [n (%)]	18 (58.1%)
16 mm, [n (%)]	1 (3.2%)
20 mm, [n (%)]	1 (3.2%)
22 mm, [n (%)]	8 (25.8%)
25 mm, [n (%)]	4 (12.9%)
31 mm, [n (%)]	3 (9.7%)
34 mm, [n (%)]	1 (3.2%)
*Device size change*^*a*^	
Smaller size, [n (%)]	1 (3.2%)
Larger size, [n (%)]	0 (0.0%)

Values are expressed as mean ± standard deviation for continuous variables and as number of patients and % for categorical variables

^a^Need for peri-procedural change to bigger or smaller device size

### Short-term results

Successful implantation of LAAO device was possible in 29 of the 31 cases (93.5%). Puncture of the interatrial septum was not possible in one patient and guiding of the implantation sheath in the LAA not possible in another patient, despite using various materials during the procedure, including a steerable sheath. These two patients were discharged without presenting any complications. There were no patients that developed a MACE, a stroke or died during or after LAAO and up to the point of discharge from the hospital. There was a single major bleeding event (3.2%) requiring a transfusion of two units of red blood cells in a patient with a hematoma and arteriovenous fistula in the vascular-access site. The arteriovenous fistula was small and did not require operative management. Three patients (9.7%) developed an acute kidney injury after the intervention, only one of them having received contrast medium during the intervention. It should be noted that this one patient only received 20 ml of contrast medium, however the duration of the interventional procedure was 100 min, the longest in our patient population. The cause of acute kidney injury in this case is presumably both pre-renal and renal (contrast medium-induced nephrotoxicity). Another patient was on diuretics and was diagnosed with a urinary tract infection during the hospital stay after the intervention (mixed pre-renal and renal etiology). In the third patient, the cause was not clear. Renal function resumed in all three patients without requiring renal replacement therapy. There were no cases of pericardial effusion or device dislocation or embolization. Short-term results after LAAO are summarized in Table 4.

**Table 4:** Short-term results after LAAO

Parameter	Study population (n = 31)
Successful implantation, [n (%)]	29 (93.5%)
MACE, [n (%)]	0 (0.0%)
Death, [n (%)]	0 (0.0%)
Major bleeding, [n (%)]	1 (3.2%)
Stroke, [n (%)]	0 (0.0%)
Vascular access-site complications, [n (%)]	1 (3.2%)
Acute kidney injury, [n (%)]	3 (9.7%)
Pericardial effusion, [n (%)]	0 (0.0%)
Device dislocation or embolization, [n (%)]	0 (0.0%)

*MACE* major adverse cardiac events

### Mid-term results

As part of the standard follow-up in all patients undergoing LAAO in our department, a follow-up visit in the outpatient unit including transthoracic echocardiography and TEE was scheduled at 3 months. After assessing available follow-up data of the n = 29 patients, in whom implantation was successful, no patients were found to have suffered a stroke. There was one case of device-related thrombus, which resolved after re-initiating and continuing oral anticoagulation in addition to clopidogrel for 9 months. One patient (3.4%) with a very big LAA treated with a 34 mm WATCHMAN FLX™ occlusion device was found to have a significant (> 5 mm) peri-device leak. One more patient had a small, non-significant leak. In one patient (3.4%), TEE revealed a persistent iatrogenic atrial septal defect at 3 months. No patients suffered a bleeding under dual antiplatelet therapy in these 3 months. Mid-term results after LAAO are summarized in Table 5.

**Table 5:** Mid-term results after LAAO

Parameter	Study population (n = 29)^a^
Stroke, [n (%)]	0 (0.0%)
Device-related thrombus, [n (%)]	1 (3.4%)
Significant peri-device leak, [n (%)]	1 (3.4%)
Iatrogenic atrial septal defect, [n (%)]	1 (3.4%)
Bleeding, [n (%)]	0 (0.0%)

^a^Only patients, in whom implantation was successful, are included in this table (29 out of 31)

## Discussion

The main finding of our study is that percutaneous LAAO is a feasible and safe stroke prevention method, alternative to oral anticoagulation, in high-risk, multimorbid patient populations with high bleeding risk. Patients having undergone TAVI prior to LAAO were included as a representative example of a patient collective with a substantial comorbidity burden. The interventional LAAO procedure was successful in most cases with low radiation burden and no (or low volumes of) contrast medium. Acute or mid-term complications were rare, and no deaths, MACEs or strokes were recorded among the patients.

The advances of interventional cardiology and the development of novel catheter-based therapies have signaled a paradigm shift in the treatment of various pathological cardiovascular conditions. Increasing age and increasing number of cardiovascular and other comorbidities result in an increased operative risk. TAVI is an excellent example of a catheter-based procedure that has given us the ability to treat patients with severe aortic valve stenosis or regurgitation, who had been traditionally inoperable. Of course, carefully identifying patients that are likely to benefit from TAVI is of major importance [11]. Risk scores, such as EuroSCORE II, that were developed to predict risk following cardiac surgery, are widely used in order to assess patient frailty [12]. Clinical judgement and a holistic baseline patient assessment are also of major importance in carefully selecting patients undergoing TAVI [11, 13]. The acknowledgement of this fact in the medical community has led to the development of interdisciplinary ‘Heart Teams’, which evaluate TAVI candidates and finally decide about the treatment option to be favored. The prevalence of pre-existing or new-onset atrial fibrillation in patients treated with TAVI has been shown to be as high as 42% [14]. Because of age and other comorbidities, almost all of these patients have a CHA_2_DS_2_-VASc score of ≥ 1 or ≥ 2 (for male and female patients, respectively), thus requiring OAC, according to current guidelines [2]. A significant number of patients being evaluated for or undergoing TAVI have an increased bleeding risk because of former bleeding events, chronic kidney disease or other contraindications to OAC. There are reports of as much as 43% of TAVI patients having a HAS-BLED score of ≥ 3 [15]. This high bleeding risk overlaps with overall frailty in these patients. For these reasons, we decided to evaluate feasibility and safety of percutaneous LAAO in patients having undergone TAVI.

A study evaluating the safety of concomitant TAVI and LAAO compared to TAVI alone was published by Attinger-Toller and co-authors in 2016 [16]. Each of the two groups included 52 patients. The primary safety endpoint at 30 days (composite of all-cause mortality and complications such as stroke, acute kidney injury, vascular complications, pericardial effusion and others) occurred in 10 patients in the concomitant group compared to 7 patients in the isolated TAVI group. Thirty-day event rates of bleeding, access-site complications and acute kidney injury were not higher in patients undergoing concomitant TAVI and LAAO. The authors concluded that “combining TAVI and LAAO is feasible and seems to be safe”.

In our study, implantation was successful in 93.5% of the patients, comparable to the reported percentages of 88–96.8% of the large, randomized trials and registries [4–6, 17]. Anatomy of the LAA and the interatrial septum were the factors not allowing successful implantation in two cases. Mean procedure duration was 55.7 min, slightly shorter than the reported mean duration in larger studies [6]. Obtaining significant experience in performing the procedure and development of new implantation materials may help to further reduce procedure duration. Guidance by TEE is an established means of imaging to visualize the fossa ovalis before puncture of the interatrial septum [3]. In our patient collective, TEE alone was sufficient to guide puncture of the interatrial septum and implantation of the occlusion device, so that no contrast medium was needed, in 67.7% of the cases. This is of major importance in patients with chronic kidney disease, who represent a significant part of our patient collective (see Table 1), because it reduces the risk of contrast-induced acute kidney injury. In the procedure of percutaneous LAAO, no contrast medium also means less fluoroscopy time, as LAA measurements are performed using TEE. Reports about mean fluoroscopy time during percutaneous LAAO are varying in the literature and range between 7 and 24 min, with techniques such as “integrated echocardiography and fluoroscopy imaging” and “shaping-the-sheath” being reported as ways to reduce fluoroscopy time [6, 18, 19]. In our study, mean fluoroscopy time was 12.4 min using the common approach recommended by the manufacturer of the occlusion device.

In our high-risk population there were no MACEs, strokes, or deaths during the intervention and until discharge from the hospital, in agreement with literature reporting such events as being uncommon [17]. Moreover, no pericardial effusions or device dislocations were observed during this period. There was one case (3.2%) of major bleeding at the vascular-access site. Sonography revealed a small arterio-venous fistula, which only required conservative management. There were three cases (9.7%) of acute kidney injury after the procedure. Only one of the three patients had received contrast medium during the intervention. None of the three patients required renal replacement therapy. In the first procedures performed in our department, contrast medium was occasionally used in addition to TEE measurements, to confirm LAA dimensions, evaluate LAA shape and, thus, optimize implantation. With increasing experience, need for contrast medium was substantially decreased. In a large registry including more than 38,000 procedures, pericardial effusions requiring intervention and major bleeding were reported to be the most common major adverse events (1.39% and 1.25%, respectively) during and after percutaneous LAAO, while device embolization was rare (0.07%) [17].

At 3 months there were no patients that had a stroke in our study population. There was a single case (3.4%) of device-related thrombus, which resolved after combined anticoagulation and clopidogrel administration for 9 months. This percentage is similar to the 3.2% described in the literature [20]. Peri-device leak could be seen in the TEE in two cases (6.9%), only one of which was significant (> 5 mm). Prevalence of peri-device leak percentage was lower than the one reported in the literature, which reached 12.5% [20].

## Limitations

Our study has some important limitations. It is a relatively small, registry-based study and has a short follow-up time. Thus, prevalence rates reported may be over- or underestimated and should be interpreted with caution. Moreover, the small size of the study and the short follow-up do not allow any conclusions about the effectiveness of percutaneous LAAO in preventing cardioembolic stroke in patients with atrial fibrillation.

## Conclusion

Literature assessing LAAO in various patient subgroups with high bleeding risk is sparse. This is the first study evaluating feasibility and safety of percutaneous LAAO in a high-risk, frail patient group with a lot of comorbidities, namely patients that underwent TAVI. Our study shows that percutaneous LAAO may represent a feasible alternative strategy for stroke prevention, that can be safely performed in high-risk, multimorbid patients with high bleeding risk or contraindications to oral anticoagulation. Although we are confident that data presented here provide some insight into the safety and efficacy of percutaneous LAAO in these patients, randomized clinical trials are needed, to be able to draw safe conclusions.

## Data Availability

Datasets generated and analyzed during the current study are not publicly available because they contain confidential information. They can be provided by the corresponding author only on reasonable request.

## Abbreviations

AF: Atrial fibrillation
GFR: Glomerular filtration rate
LAA: Left atrial appendage
LAAO: Left atrial appendage occlusion
MACE: Major adverse cardiac events
NOACs: Newer, non-vitamin K oral anticoagulants
OAC: Oral anticoagulation
TAVI: Transcatheter aortic valve implantation
TEE: Transesophageal echocardiography
